# Asymmetric Effects of Quality of Life on Residents’ Satisfaction: Exploring a Newborn Natural Disaster Tourism Destination

**DOI:** 10.3390/ijerph182111577

**Published:** 2021-11-04

**Authors:** Derong Lin, Zuoming Jiang, Hailin Qu

**Affiliations:** 1 School of Management, Xiamen University, Xiamen 361005, China; drlin65@xmu.edu.cn (D.L.); h.qu@okstate.edu (H.Q.); 2 School of Hospitality and Tourism Management, Spears School of Business, Oklahoma State University, Stillwater, OK 74078, USA

**Keywords:** attributes of quality of life, residents’ satisfaction, asymmetric effect, newborn natural disaster destination, Three-Factor Theory

## Abstract

Developing disaster tourism has become an important means of post-disaster recovery, but the relationship between attributes of quality of life (QOL) and residents’ satisfaction toward newborn natural disaster destination (NNDD) development has not been explored. This study examines the asymmetric effects of QOL on residents’ satisfaction using the dummy variable regression approach based on the Three-Factor Theory. A mixed-method approach was used to develop QOL scale, and a questionnaire was developed to survey 379 residents from China’s Wenchuan. This study identifies the types (positive asymmetric, symmetric, and insignificant effect) of seven QOL attributes that influence residents’ satisfaction. This study provides a valuable supplement to the literature on residents’ QOL by focusing on the NNDD context, and is a pioneering attempt to apply the Three-Factor Theory from the perspective of local residents. Practically, some implications for policy optimization are proposed to help NNDD development.

## 1. Introduction

Natural disasters have always accompanied the development of human society. An important means of promoting post-disaster recovery is to excavate disaster site resources to develop tourism [1,2]. The term newborn natural disaster destinations (NNDD) refers to natural disaster heritage sites or artificial disaster memorial spaces that attract tourists. As a newly built destination after a natural disaster, it functions as a space to mourn compatriots and carry out patriotism education, earthquake prevention education, and scientific research [3]. Recently, natural disaster tourism research has gained increased attention [4,5]; however, there remains a lack of an effective indicator to measure the recovery effect of post-disaster destinations. Given the imbalance between disaster tourism development and theoretical research greater exploration of the NNDD context is needed.

This study’s focal NNDD, the Wenchuan disaster tourism community, experienced an 8.0-magnitude earthquake in China on 12 May 2008 [6]. The Wenchuan NNDD is located in Wenchuan County, Aba Prefecture, Sichuan Province, and includes the epicenter of Yingxiu, Shuimo ancient town, and Sanjiang ecological area. The government has identified tourism as one of the leading industries for post-disaster reconstruction after considering the needed economic recovery and complex geological conditions. This is the first time that a plan for reconstructing and developing the tourism industry has been incorporated into the national plan for post-disaster recovery and reconstruction, innovating the development of natural disaster tourism in China [7]. Thirteen years on, the Wenchuan disaster tourism community has been named the best example of post-disaster reconstruction by the United Nations [8] and the most scenic spot in China [9]. However, this community has entered a degenerating stage after a decline in tourist quantity since 2015. So, it is necessary to further investigate and improve the sustainable development ability of NNDDs to provide suggestions for local government policy optimization.

At present, limited studies have observed the evolution trend of NNDDs [10]. Wright and Sharpley (2018) suggested that natural disaster tourism is temporary, with the completion of post-disaster reconstruction and the decline of social attention, disaster tourist numbers would significantly decrease [11]. If the downward trend continues, it will affect the residents’ QOL (their living standards and living environments in the NNDD development process). Simultaneously, residents’ dissatisfaction will exacerbate the vicious cycle. Thus, policymakers must scientifically evaluate residents’ satisfaction toward NNDD development according to their QOL attributes. However, no literature comprehensively measures residents’ QOL attributes in the NNDD context and compares the differences between conventional destinations and NNDDs. The possible reason for this deficiency is the lack of a measurement tool. Therefore, this study attempts to strengthen our understanding of residents’ QOL attributes by developing a new scale in the NNDD context.

Residents’ QOL attributes is an important indicator to evaluate the effect of NNDD development policy [12]. If development is good, it can achieve economic renewal, social restructuring, and cultural renaissance [13]. Recently, the positive effect of residents’ QOL attributes (material/non-material domains) on tourism development satisfaction of conventional destinations has been confirmed, and vice versa [14]. However, existing literature neglects to evaluate the NNDD development satisfaction from the perspective of residents’ QOL attributes. Thus, this study adopts scientific methods to explore asymmetric effect relationships between QOL attributes and residents’ satisfaction.

**Figure 1:**
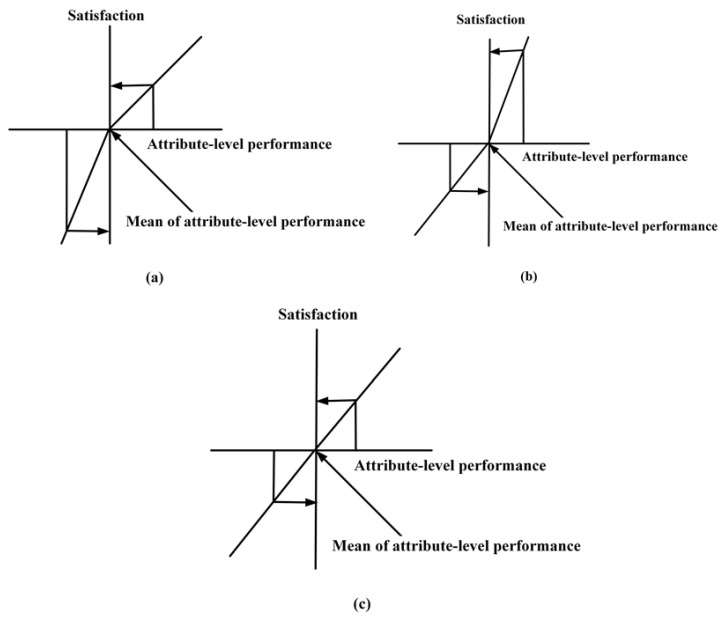
(**a**) Negative asymmetric effect; (**b**) Positive asymmetric effect; (**c**) Symmetric relationship. Notes: Adapted from references [18].

Recently, researchers have proposed that the relationship between destination attributes and tourist satisfaction is asymmetrical [15,16]. Asymmetric effects refers to two attributes of above and below the expectation level have different effects on tourist satisfaction. This perspective breaks through the limitations of the underlying assumption of a symmetric relationship between destination attributes and tourist satisfaction. Previous studies divide destination attributes according to different asymmetric effects into basic, excitement, and performance factors based on the Three-Factor Theory [15,17]. The three-factor theory is an effective classification assessment method in tourist satisfaction. Specifically, the basic factor attributes will lead to dissatisfaction when they fail to meet tourist needs, but, even if these attributes can fully meet their needs, they will not cause tourist satisfaction. This is called a negative asymmetric effect (Figure 1a) [18]. On the other hand, the excitement factor attributes will lead to satisfaction when they meet tourist needs, but, even if these attributes cannot fully meet tourist needs, they will not cause tourist dissatisfaction. This is called a positive asymmetric effect (Figure 1b) [18]. Finally, the performance factor attributes will lead to satisfaction when they meet tourist needs, but, dissatisfaction when they fail to meet those needs. This is called a symmetric effect (Figure 1c) [18]. The Three-Factor Theory were used to assess the asymmetric effects between luxury hotel services, urban tourism attributes, and rock-climbing destination attributes and tourist satisfaction [19,20,21], these asymmetric results provide reliable evidence for destination policy optimization. Therefore, the good practice of the Three-Factor Theory provides a strong enough justification for this study.

Natural disaster tourism is considered a complex and unique experience. Zhang (2021) identified six main dimensions of experience: cognitive, emotional, introspective, sensory, relational, and hedonic [3]. Therefore, tourists’ satisfaction is to search the meaning of life from the perspective of death. It is well-known that destinations’ sustainable development must consider both tourists’ and residents’ interests [22,23]. Compared with the literature on tourist satisfaction, resident satisfaction evaluations are lacking. As a special destination type, the NNDD satisfaction evaluation should focus on residents’ QOL. Is there an asymmetric effect relationship between QOL attributes and residents’ satisfaction? If so, how would different QOL attributes be classified? To address this gap, this study selected Wenchuan NNDD to explore the asymmetric effect. The research objectives are (1) to identify the residents’ QOL attributes in the NNDD; (2) to test the asymmetric effects of QOL attributes on residents’ satisfaction; and (3) to optimize residents’ satisfaction policies according to different attribute factor categories.

## 2. Literature Review

### 2.1. Residents’ QOL Attributes in NNDDs

Destination residents’ QOL is defined as “tourism destination residents’ cognitive judgments and emotional responses toward various life domains” [24] (p. 57). Presently, research measuring residents’ QOL mainly examines its multi-attribute nature (a combination of material and non-material domains). Kim et al. (2013) identified four QOL attributes: material well-being, community well-being, emotional well-being, and health and safety well-being [25]. Liang and Hui (2016) further developed six QOL attributes: urban issues, economic strength, family and personal well-being, community well-being, way of life, and community awareness and facilities [24]. Andereck and Nyaupane (2011) proposed eight QOL attributes using a new measurement method, including community well-being, urban issues, way of life, community pride and awareness, natural/cultural preservation, economic strength, recreation amenities, and crime and substance abuse [26]. These studies provide valuable references for this one.

An NNDD is a place where tourists go to visit natural disaster heritage sites or artificial disaster memorial spaces. As tourist attractions, NNDDs become objects of the “natural disaster tourism” gaze and community residents’ QOL is greatly affected by the development of disaster tourism. Existing studies mainly highlight a single residents’ QOL attribute domain, such as housing reconstruction, transportation networks, physical and mental health, and community relations [9,27,28]. Thus, understandings of residents’ QOL in the NNDD context are limited and a multi-attribute scale for measuring residents’ QOL remains to be developed. So, we develop a new scale based on previous QOL scales, resident interviews, and the NNDD context.

### 2.2. Residents’ Satisfaction toward NNDD Development

As with Phoenix tourism defined by Miller et al. (2017), NNDD development is a process of destination regeneration, newborn, and revitalization after a natural disaster [13]. Once a natural disaster site becomes a tourism destination, residents’ living environments are positively and negatively affected. Scholars have focused on natural disaster tourism patterns and their effects on residents’ satisfaction [29,30]. Kato (2018) suggests that natural disaster tourism patterns should be designed considering local socio-cultural, political, economic foundations, and the natural environment [31]. “Community-based tourism pattern” offers a “win-win” situation and is recognized by residents and tourism industry managers [32].

Every major natural disaster has caused huge losses and had far-reaching impacts. NNDD development is complex and long-lasting; thus, residents’ attitudes towards NNDD development have stages. In the early material reconstruction stage, scholars focus on the macro policy satisfaction. NNDD planning considers both the current recovery and reconstruction and the long-term development and upgrading [33]. Later, in the spiritual reconstruction stage, scholars measure the micro-cultural psychology of residents [8,34]. Thus, residents’ satisfaction towards NNDD development includes reconstruction of the material life and the spiritual home. There are many studies on residents’ attitudes and their characteristics of satisfaction, while the asymmetric influencing factors of residents’ satisfaction lack exploration. Addressing this, we conduct a correlation study between the QOL attributes and residents’ satisfaction to identify the influencing factors and internal laws.

### 2.3. The Relationship between QOL Attributes and Residents’ Satisfaction

It is common knowledge that sustainable tourism development needs to consider and meet local residents’ living standards [35]. Currently, there is no research on the relationship between QOL attributes and residents’ satisfaction towards NNDD development. However, social exchange theory can provide a reasonable explanation for this relationship. If residents perceive a higher QOL, given the psychological exchange, they are satisfied with NNDD development. Conversely, if they perceive a low QOL, they will oppose or passively resist the implementation of NNDD development policies. Moreover, according to the asymmetric effect [36], the effect of QOL attributes on residents’ satisfaction may be asymmetrical. Some attributes can satisfy residents and have competitive advantages, while others may cause residents dissatisfaction and need to be further improved.

Most previous studies use linear regression or structural equation models to measure residents’ satisfaction [14,24]. These methods accept that there is a linear relationship between QOL attributes and residents’ satisfaction. The attribute weight is determined by the regression coefficient—the larger the regression coefficient, the more important the attributes. However, the assumption of linear relationship ignores the different relationship models of different attributes on residents’ satisfaction [37,38]. Therefore, there may be a non-linear/asymmetric relationship between QOL attributes and residents’ satisfaction. Subsequently, this study applies the Three-Factor Theory to explore the asymmetric relationship.

### 2.4. The Three-Factor Theory of Customer Satisfaction

Kano et al.’s (1984) Three-Factor Theory can fully understand the asymmetric effect between attribute performance and customer satisfaction [39]. Regarding the asymmetric effect on satisfaction, research has divided product attributes into three categories: basic factors, excitement factors, and performance factors [40]. As stated in the introduction section, basic factors are necessary but not sufficient conditions for customer satisfaction, because in the customer’s eyes, the basic factors are taken for granted; on the contrary, excitement factors are sufficient but unnecessary conditions for customer satisfaction, because the excitement factors are outside the expectation of customer demand, they can provide unexpected surprises; performance factors are the necessary and sufficient conditions for customer satisfaction, because there is a symmetric relationship between performance factors and customer satisfaction [39].

The asymmetric effects of tourist satisfaction have been confirmed by previous studies in different destinations, including sun and sand destination [37], pilgrimage destination [41], urban tourism destination [21], and rock-climbing destination [19]. Researchers have recognized the importance of distinguishing three factors (basic, performance, and excitement factors) in order to manage tourist satisfaction more effectively. However, there is still a lack of literature on resident satisfaction based on the Three-Factor Theory. So, we fill this gap in the NNDD context.

## 3. Methods

A mixed-method approach was used with an exploratory qualitative study (phase one: literature review, residents interviews, and expert panel discussion) to develop the measurement scale and ensure the validity and reliability of the instrument. Then, a quantitative study (phase two: survey and modelling) assessed the asymmetric effects of QOL on residents’ satisfaction.

### 3.1. Phase One: Qualitative Study

First, the initial scale items were derived from reviewing the previous literature on residents’ QOL in conventional destinations. Simultaneously, we considered the characteristics of the NNDD and post-disaster reconstruction plans issued by the government. Reconstruction plans include residential housing, infrastructure, public service facilities, industrial development, and ecological restoration, which are closely related to residents’ QOL. This study principally considered the literature on the following eight aspects: housing conditions, work income, life facilities, medical care and education, family life, health and safety, social relations, and entertainment opportunities. Based on these, we extracted and amended 23 related scale items (Table 1); then, we constructed eight residents’ QOL dimensions. These QOL domains can be divided into material (one to four) and non-material (five to eight) groups. NNDD development is a systematic project, involving every aspect of the residents’ life; the measurement scale not only includes the newly built housing, hospitals, schools, and other material aspects but also covers family life, social relations, and other non-material aspects. Based on the content analysis of post-disaster reconstruction planning, this study verified eight residents’ QOL constructs with 20 related scale items (Table 1).

Second, the items of the scale were further developed and identified by interviewing local residents. Eight residents of Yingxiu town were invited to participate in the study (four male, four female), the number of interviewees was determined by the degree of information saturation, and the interview process ended when the interviewees had no new insights emerged [43]. A semi-structured interview was organized around the following open questions: (1) Could you share the impact of developing disaster tourism on your life after the Wenchuan earthquake? (2) What aspects are you satisfied with and not satisfied with in your living conditions? (3) In order to improve residents’ happiness, what suggestions do you have for the development of the Wenchuan disaster destination? The length of the interviews each lasted about an hour. Most of the content was consistent with the literature review—work income (e.g., enough good jobs for residents), medical care and education (e.g., the government provides free employment skills training), and social relation (e.g., harmonious neighborhood). However, some new items emerged, such as residential architecture highlights local culture, exchange business experience, and marketing restaurants online. Forty items were identified through the literature review and residents’ interviews (Table 1). For example, the following interviewee 3 statement indicates the domain “work income”.

I started my restaurant seven or eight years ago, and at that time, just after the earthquake, there were not many other jobs, and the medicine business was not good. After seeing more tourists come, the tourism development improved, and I opened the restaurant. However, now there are fewer tourists, and business is not good. [Interviewee 3]

Third, an expert panel discussion was organized to help ensure the content validity of the scale items in April 2019. The panel members included three government officials from Yingxiu town and three tourism sociology professors. The experts were informed of the discussed subject (scale development), the research sample (NNDD residents), and the survey region (Wenchuan County). They reviewed and assessed the representativeness and rationality of the measurement items with 1 as strongly inapplicable and 5 as strongly applicable. The experts could also provide comments and suggestions. Consequently, eight items with low ratings, such as high community wage, plenty of parks and open space, and environmental cleanliness (air, water, soil), were deleted. Therefore, 32 items were retained.

Finally, a pilot survey was conducted to test the reliability and validity of the scale items. Data were collected in Yingxiu town in June 2019; each household was issued a questionnaire, and a total of 163 valid questionnaires were collected. The results of EFA and Cronbach’s Alpha showed that the eight domains were significantly differentiated. The Alpha scores higher than 0.7 (0.702–0.829) indicated that the structure of the scale was reliable and valid [44].

### 3.2. Phase Two: Quantitative Study

The questionnaire included three sections. Section 1 investigated residents’ QOL perceptions in Wenchuan NNDD. The measurement scale included eight domains that emerged, comprising 32 items. Section 2 included three measure items of residents’ satisfaction, “I was satisfied with the NNDD development,” “I was pleased with the change from NNDD development,” “I had confidence in the future of NNDD development.” Respondents were asked to express their perceptions using a five-point Likert scale with 1 set as “totally disagree” and 5 setting as “totally agree.” Section 3 deals with respondents’ demographics including gender, age, education, occupation, monthly income, and length of residence.

#### 3.2.1. Sampling and Data Collection

The survey data were collected in Wenchuan County from July to August 2019. The survey sites were Yingxiu, Shuimo, and Sanjiang. First, two streets and one square were randomly selected from each town using the probability-proportional-to-size sampling method, and a total of six streets and three squares constituted sampling units [45]. Second, we used the systematic sampling method to select 50 families in each street and 50 residents in each square [46]. One family member was selected from every 10 families in the street, and one resident was selected from every 20 people in the square to participate in this study. Only respondents aged at least 28 years old (all adults when the 2008 Wenchuan earthquake occurred) were selected to improve the representativeness and the QOL perception of the sample surveyed. The survey team comprised undergraduates from the Aba Normal University in Sichuan province and the authors of this study, which was convenient for language communication with local residents of the Qiang, Tibetan, and other ethnic minorities. A total of 450 questionnaires were distributed to residents, 71 invalid questionnaires were eliminated due to missing information and apparent lack of earnest response, so 379 valid questionnaires were obtained.

#### 3.2.2. Data Analysis

Firstly, exploratory factor analysis was conducted, and the items were deleted to identify the basic domain of residents’ QOL attributes [44]. Secondly, confirmatory factor analysis was conducted to confirm the domain division of the attributes and the reliability, validity, and fitting degree of the data were comprehensively tested [47]. Finally, researchers generally prefer to use the dummy variable regression method to analyze the asymmetric effects [48,49]. A dummy variable regression was used to test the asymmetric effect between QOL attributes and residents’ satisfaction [40]. In the first step, the results of the confirmatory factor analysis were used to calculate the average value of the sum of the measured items in each domain as the attribute score. The second step was to convert the scores into dummy variables and calculate the tertiles of each attribute score. High scores higher than or equal to 66.66% were converted to (1,0); low scores lower than or equal to 33.33% were converted into (0,1); the average score was converted into (0,0). Thus, a regression model with multiple dummy variables was constructed.

## 4. Results

### 4.1. Demographic Information

According to demographic characteristics (Table 2), there were more females (52.5%) than males (47.5%). Most respondents were young and middle-aged; ages 28–55 accounted for 78.1%. Regarding education, 83.9% held a secondary school degree or below. For length of residence, 65.4% had been living there for more than 20 years. In terms of income, 32.5% earned less than ¥1000, 35.6% earned from ¥1000–2000. Most respondents were farmers (34.8%) or owned individual businesses for tourism (20.1%).

The result of Alpha of 32 measure items was 0.902, indicating high internal consistency and reliability among items [44]. The *KMO* value was 0.835 and the Bartlett spherical test was significant (*Chi-Square* = 3050.312, *df* =4 96, *p* < 0.001), indicating that the scale was suitable for factor analysis [44]. Then, the samples were randomly divided into two subsamples for exploratory factor analysis and confirmatory factor analysis (sample 1, *n* = 189; sample 2, *n* = 190).

### 4.2. Exploratory Factor Analysis

An exploratory factor analysis was conducted to determine the dimensions of the scale items, using principal component analysis with maximum variance rotation. The following principles are followed to extract common factors: first, the eigenvalue is greater than or equal to 1; second, the item is deleted when the factor load after rotation is less than 0.5 or more than 0.4 on multiple factors at the same time [44]. After three cycles of factor analysis, 6 items were deleted, and 26 items were retained. The results showed that the cumulative variance contribution rate of the seven factors = 67.338%, which is larger than the acceptance standard of 60% [44]. The *KMO* was 0.826. The results of Alpha of all seven factors were above 0.7, indicating that the data quality was reliable [44]. According to the items contained in each factor, the seven factors were named “work income (WI),” “social relations (SR),” “housing conditions (HC),” “medical conditions (MC),” “entertainment opportunities (EO),” “family life (FL),” and “education conditions (EC)” (Table 3). Compared with the original eight scale dimensions of QOL attributes, the dimensions of “life facilities” and “healthy and safety” were not supported by data, and the “medical and education” dimension was decomposed into two attribute factors of “medical conditions” and “educational conditions.”

### 4.3. Confirmatory Factor Analysis

Given that all scale constructs were measured by self-management reports, common method bias (CMB) was considered as a serious concern. Therefore, we conducted Harman’s single-factor analysis to examine CMB, which is among the most widely utilized techniques for testing the potential threat of CMB [50]. Seven factors with eigenvalues greater than 1 emerged; the total variance of a single factor was 27.948% (<50%). These results indicate that the data were unbiased against CMB [50].

The reliability and validity of the multidimensional structure were measured by confirmatory factor analysis. The fit indexes of the initial measurement model showed that the model did not perform well (*χ*^2^/*df* = 2.236, GFI = 0.795, IFI = 0.85, CFI = 0.847, RMSEA = 0.081). According to the previous research methods [46], we deleted three items with low factor loading values (less than 0.5) to improve the goodness of fit. The model was modified by a Modification Index to reduce the value of *χ*^2^. The fit indexes of the new model structure were *χ*^2^/*df* = 1.948, GFI = 0.84, IFI = 0.907, CFI = 0.905, and RMSEA = 0.071. Thus, the goodness of fit was improved and met the criteria [43]. As shown in Table 3, the factor loadings reached significant levels for all items, greater than 0.5 (0.570–0.995). The individual item reliability (R^2^) of all structures was greater than 0.3. The composite reliability of the model greater than 0.7 (0.758–0.868). The AVE of most constructs was greater than 0.5; only “family life” was slightly lower at 0.4412. As it was close to 0.5, which was retained so as not to affect subsequent analyses. Generally, the internal validity of the model was good. Table 4 shows that the square roots of AVE of all dimensions were greater than the correlation coefficient among all dimensions, indicating that the measurement model had good discriminant validity [44].

### 4.4. Asymmetric Effects Analysis

According to the steps described above [40], the scores of each attribute were converted into dummy variables to build a regression model. We used the tertiles of the attributes to distinguish between high, average, and low scores. This study defined “low performance” (0,1) as all scores lower than or equal to the former threshold value (33.33% tertile), “high performance” (1,0) as those higher than or equal to the latter threshold value (66.66% tertile), and “average performance” (0,0) as those falling between the two threshold values. For each attribute, two regression coefficients were obtained, one to measure the effect when the score was low, the other when the score was high. Subsequently, a regression model with multiple dummy variables was constructed as follows:

(1) $$\begin{array}{l} ROS=Cons\tan t+\beta_{1}H\_WI+\beta_{2}L\_WI+\beta_{3}H\_SR+\beta_{4}L\_SR+\beta_{5}H\_HC+\\ \beta_{6}L\_HC+\beta_{7}H\_MC+\beta_{8}L\_MC+\beta_{9}H\_EO+\beta_{10}L\_EO+\beta_{11}H\_FL+\\ \beta_{12}L\_FL+\beta_{13}H\_EC+\beta_{14}L\_EC+error \end{array}$$

where *ROS* = the score for residents overall satisfaction and *H_WI*, *H_SR*, *H_HC*, *H_MC*, *H_EO*, *H_FL*, *H_EC* = high scores for work income attribute, social relations attribute, housing conditions attribute, medical conditions attribute, entertainment opportunities attribute, family life attribute, and education conditions attribute, respectively. *L_WI*, *L_SR*, *L_HC*, *L_MC*, *L_EO*, *L_FL*, *L_EC* = low scores for the same respective attributes.

The standardized regression coefficients cannot be clearly and reasonably explained in dummy variable regression, due to the risk of interpretation bias risk. As per Mikulić and Prebežac (2012), the results of non-standardized regression coefficients were used in regression analyses [51]. The dummy variable regression model explained 50.6% of the variance of the overall satisfaction (*F* = 28.704, *p* < 0.001) and eight out of the fourteen independent variables’ coefficients were significant. Besides, the VIF values of all variables were below 10, and the multicollinearity problem was controlled (Table 5).

After comparing the absolute value of the dummy variable coefficient of the high scores and low scores of each attribute, the QOL attributes had an asymmetric effect on residents’ satisfaction. The regression coefficients of “social relations,” “housing conditions,” “medical conditions” and “education conditions” attributes were significant when the score was high but not when the score was low. This indicated that these four attributes had a stronger influence on residents’ satisfaction when the score was high (a positive asymmetric effect). Such attributes were classified as excitement factors for residents’ satisfaction. The regression coefficients of the “entertainment opportunities” and “family life” attributes were significant when the score was high or low. Therefore, there was a symmetric relationship between the two attributes and residents’ satisfaction, and these were classified as performance factors. It should be noted that “work income” was an unimportant excitement factor, although the regression coefficient in the high score was greater than the low score, the two coefficients were statistically insignificant [51]. Thus, the assumption of a linear relationship ignores the varying effects of different attributes on residents’ satisfaction.

## 5. Discussion and Implication

### 5.1. Theoretical Implications

This study presents three theoretical contributions. First, it measured the differences in residents’ QOL attributes between conventional destinations and NNDDs. Previous studies have divided the residents’ QOL attributes in conventional destinations into four domains, six domains and eight domains [24,25,26]. This study developed a scale to measure the residents’ QOL attributes in NNDDs; the results of EFA and CFA contain 23 measurement items in seven domains. The four domains of “work income,” “family life,” “social relations,” and “entertainment opportunities” were consistent with the conventional destination research conclusions, which indicated that residents also pay attention to these domains in NNDDs, but their contents were marked by post-disaster reconstruction and development. On the other hand, “housing conditions,” “education conditions,” and “medical conditions” were important domains of residents’ perceptions. The results show that the reconstruction of material life was a distinguishing feature. Thus, this study provides the first scale to measure residents’ QOL in NNDDs, laying a good foundation for further study.

Second, this study confirmed the existence of symmetric and asymmetric effect factors of QOL attributes on residents’ satisfaction. The results show that the QOL attributes affect the residents’ satisfaction in different ways. Of the seven attributes, only “family life,” “entertainment opportunities,” and residents’ satisfaction expressed a symmetric relationship. The other four attributes of “social relations,” “housing conditions,” “medical conditions,” and “education conditions” expressed a positive asymmetric relationship. “Work income’s” insignificant effect may be related to the NNDD development stage of the case study. After nearly a decade, tourist numbers in Wenchuan dropped from a peak of 5.71 million in 2015 to 3.1 million in 2018, affecting the job opportunities of residents [9]. Our results show that only 28.5% of the respondents were engaged in jobs directly related to tourism, possibly explaining why “work income” was not significant to residents’ satisfaction.

Third, the application of the Three-Factor Theory was extended from destination tourists to residents. It has been widely applied to the study of tourist satisfaction in different tourism destinations [15,16,17]; however, no studies investigate residents’ satisfaction using this theory. There is a consensus that the premise of sustainable development of tourism destination is to satisfy both tourists and residents [22]. The QOL satisfaction of residents is an important guarantee for NNDDs’ sustainable development [32]. Therefore, our results further support the pioneering application of the Three-Factor Theory in measuring residents’ satisfaction.

### 5.2. Practical Implications

An in-depth analysis of the asymmetric effect of the QOL attributes can provide suggestions for local governments to optimize NNDD development policies. The regression results of dummy variable showed that performance and excitement factors attributes should be optimized toward residents’ satisfaction.

First, two performance factors attributes should be continuously improved to maintain the stability of high satisfaction. Since there was a symmetric relationship between the performance factors attributes and residents’ satisfaction, this effect on satisfaction was the most stable. Therefore, the government should play into the driving effect of tourism, combining NNDD development with spiritual homeland and socio-cultural reconstruction to improve tourism’s social value. More entertainment facilities and cultural squares should be built to allow residents to partake in more family activities.

Second, four excitement factors attributes should be effectively promoted to create more enthusiasm. Excitement factors attributes were generally not expected as residents have no clear demand for them. However, these NNDD development policies should be enhanced to create pleasant life surprises. Here, local government can obtain residents’ opinions and suggestions through WeChat, microblog, telephone, and field surveys to identify high demand items. Moreover, the government should set up NNDD sustainable development monitoring stations and entrust university research teams as third parties to carry out residents’ QOL assessments regularly. Additionally, policies (town construction, public services, and festival activities) should be introduced to enhance residents’ sense of community belonging.

Third, “work income” as an unimportant excitement factor attribute should be continuously monitored so that it does not harm residents’ satisfaction. Economic development was the material basis for improving residents’ QOL; after a period of leapfrog development, with tourist numbers decreasing, some NNDDs have entered a recessionary adjustment phase. Therefore, local industrial structures should be optimized to encourage the development of indigenous planting, animal breeding, and ethnic handicraft industries to provide more job opportunities and improve residents’ income.

## 6. Conclusions

Following a rigorous step in previous studies on scale development and validation, the study established the validity and reliability of the QOL scale. Specifically, using EFA, with the seven scale dimensions of QOL attributes, 26 items were retained in the identified scale. The construct dimension and measurement scale were further verified by CFA. The scale provides a valuable supplement to the literature on residents’ QOL by focusing on the NNDD context.

**Figure 2:**
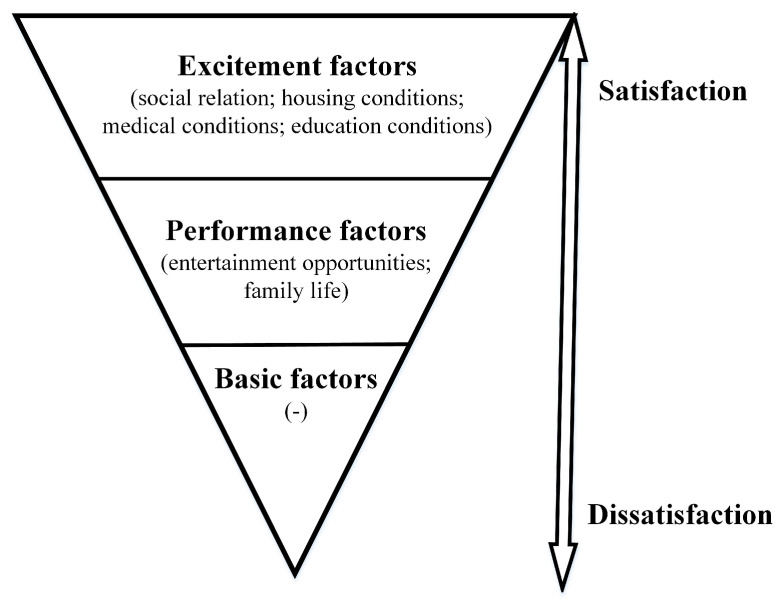
Optimization attributes of QOL for residents’ satisfaction. Notes: “work income” was an unimportant excitement factor attribute; “-” represent there is no relevant attribute in the basic factor.

Figure 2 shows the classification and prioritization of QOL attributes as an inverted pyramid structure. The higher the residents’ satisfaction, the greater the quantity of QOL attributes. The results of this study divided the QOL attributes into performance and excitement factors. There was no relevant attribute in the basic factor category, indicating that the residents have a high degree of satisfaction, and the “people-centered” NNDD development policy has met their basic needs.

This study has some limitations. It provides a measurement scale of residents’ QOL in NNDDs in China; however, the scale model has not been examined in other countries or regions with different populations. Additional research needs to test the validity of the scale in other countries that have developed disaster tourism such as Japan. Analyzing only cross-sectional data of multiple NNDDs is another limitation of the study. Since residents’ satisfaction is a dynamic process, a longitudinal study with multilevel subjects should be conducted to continually monitor the QOL attributes changes and residents’ new expectations. Finally, the “work income” dimension was eliminated because of its insignificant effect on residents’ satisfaction, which may have led to information loss. Considering that “work income” is a fundamental factor for residents’ QOL, future research should continue to focus on it.

## Figures and Tables

**Table 1:** Scale items derived from literature, NNDD context, and resident interviews.

QOL Domains	Scale Items	References	NNDD Context	Resident Interview
Housing Conditions	Moved into a new house		√	√
	Home was spacious			√
	Well-equipped facilities (water, gas, electricity)	[42]		√
	Residential architecture highlights local culture			√
	Housing function combination commercial and residential		√	√
Work Income	Enough good jobs for residents	[24,25,42]		√
	Selling local specialty products		√	√
	Stores and restaurants owned by residents	[24,35]	√	√
	Explaining disaster heritage and memorial halls		√	√
	High community wage	[24]		
Life Facilities	Public transportation to and from other communities	[26,42]	√	√
	Roads, bridges, and utility services facilities	[24,35]	√	√
	Shopping facilities	[42]		
	Plenty of parks and open space	[24]		√
	Exercising facility			√
Medical and Education	Community hospital facilities were advanced	[24,42]	√	√
	Medical supplies were adequate	[42]		√
	Going to the hospital was convenient			√
	New schools were stronger		√	√
	The government provides free employment skills training	[42]	√	√
Family Life	Family relationships were harmonious	[24]	√	√
	Family ties were stronger		√	√
	Family members take care of each other		√	√
	More time to exercise	[25]		√
	Life was relaxed and happy	[25,26]		√
Health and Safety	Environmental cleanliness (air, water, soil)	[26,42]		√
	The beauty of my community	[24]		√
	The earthquake resistance level of the building was improved		√	√
	The mountain was strengthened		√	√
	The flood levee was reinforced		√	√
Social Relations	Harmonious neighbourhood	[24,42]	√	√
	Opportunities to be with friends and relatives	[42]	√	√
	Opportunities to interact with tourists	[24]	√	√
	Exchange business experience			√
	Selling restaurants online			√
Entertainment Opportunities	Publicly funded recreation	[42]	√	√
	Plenty of festivals, fairs	[26]		√
	Spending on entertainment increased			√
	More entertainment activities	[35]		√
	Having sports events to participate in in my community	[26]		√

Notes: ‘√’ indicates the existence of this source.

**Table 2:** Demographics of respondents (*n* = 379).

Profile	Category	%	Profile	Category	%
Gender	Male	47.5	Monthly income	Less than RMB 1000	32.5
	Female	52.5		RMB 1000–2000	35.6
Age	28–35	5.3		RMB 2001–4000	21.9
	36–45	31.9		More than RMB 4000	10.0
	46–55	40.9	Occupation	Individual businesses for tourism	20.1
	>55	21.9		Tourism workers	8.4
Education	Junior high school or below	58.8		Transportation personnel	1.8
	High school or technical secondary school	25.1		Farmers	34.8
	Junior college	13.2		Retired	6.3
	Undergraduate or above	2.9		Teachers	8.7
Length of residence	<5 years	14.2		Personnel of enterprises and institutions	6.6
	5–10 years	7.4		Other	13.2
	11–20 years	12.9			
	>20 years	65.4			

**Table 3:** Results of factor analysis.

Dimensions of Attributes	Items of Scale	Exploratory Factor Analysis (N = 189)			Confirmatory Factor Analysis (N = 190)			
		Factor Loading	Eigenvalue (% of Variance)	Cronbach’s Alpha	Factor Loading	Individual Item Reliability (R^2^)	Composite Reliability	AVE
Work Income	Enough good jobs for residents	0.768	7.101 (12.185%)	0.817	0.711	0.506	0.837	0.5639
	Selling local specialty products	0.764			0.826	0.682		
	Stores and restaurants owned by residents	0.737			0.791	0.625		
	Explaining disaster heritage and memorial halls	0.705			0.665	0.443		
Social Relations	Harmonious neighborhood	0.771	2.973 (11.913%)	0.809	0.862	0.743	0.8353	0.5646
	Opportunities to be with friends and relatives	0.756			–	–		
	Opportunities to interact with tourists	0.707			0.721	0.519		
	Exchange business experience	0.699			0.570	0.325		
	Selling restaurants online	0.533			0.819	0.671		
Housing Conditions	Moved into a new house	0.736	2.309 (9.996%)	0.792	0.796	0.633	0.847	0.5814
	Well-equipped facilities (water, gas, electricity)	0.730			0.742	0.550		
	Residential architecture highlights local culture	0.666			0.814	0.662		
	Housing function combination commercial and residential	0.659			0.692	0.478		
Medical Conditions	Community hospital facilities were advanced	0.828	1.630 (9.225%)	0.826	0.873	0.762	0.8677	0.6888
	Medical supplies were adequate	0.808			0.907	0.823		
	Going to the hospital was convenient	0.791			0.694	0.481		
Entertainment Opportunities	Publicly funded recreation	0.755	1.290 (8.396%)	0.714	0.604	0.365	0.7985	0.6774
	Spending on entertainment increased	0.685			–	–		
	More entertainment activities	0.642			0.995	0.990		
	Having sports events to participate in in my community	0.636			–	–		
Family Life	Family relationships were harmonious	0.754	1.162 (8.315%)	0.711	0.629	0.396	0.7584	0.4412
	Family ties were stronger	0.697			0.674	0.454		
	Family members take care of each other	0.560			0.740	0.548		
	Life was relaxed and happy	0.509			0.606	0.367		
Education Conditions	New schools were stronger	0.839	1.058 (7.365)	0.789	0.807	0.651	0.764	0.6182
	The government provides free employment skills training	0.810			0.765	0.585		

Notes: ‘–’ indicates the removed scale item.

**Table 4:** Test of discriminant validity of measurement model (N = 190).

Variable	WI	SR	HC	MC	EO	FL	EC
WI	**0.751**						
SR	0.241 **	**0.751**					
HC	0.319 **	0.504 **	**0.762**				
MC	0.201 **	0.178 *	0.419 **	**0.829**			
EO	0.405 **	0.328 **	0.201 **	0.247 **	**0.823**		
FL	0.284 **	0.473 **	0.466 **	0.336 **	0.383 **	**0.664**	
EC	0.116	0.287 **	0.346 **	0.526 **	0.143 *	0.396 **	**0.786**

Notes: The bold diagonals are the square roots of AVEs; * *p* < 0.05, ** *p* < 0.01.

**Table 5:** Results of the regression with dummy variables (N = 379).

Variables of Attributes	Non-Standardized Regression Coefficients	SE	t-Value	*p*-Value	VIF
H _ Work Income (H _ WI)	0.114	0.085	1.348	0.179	1.322
L _ Work Income (L _ WI)	0.022	0.056	0.397	0.692	1.350
H _ Social Relations (H _ SR)	0.129 *	0.053	2.460	0.014	1.280
L _ Social Relations (L _ SR)	−0.215	0.121	−1.776	0.077	1.172
H _ Housing Conditions (H _ HC)	0.147 **	0.056	2.624	0.009	1.446
L _ Housing Conditions (L _ HC)	−0.096	0.114	−0.844	0.399	1.144
H _ Medical Conditions (H _ MC)	0.196 ***	0.055	3.554	0.000	1.384
L _ Medical Conditions (L _ MC)	−0.102	0.093	−1.102	0.271	1.375
H _ Entertainment Opportunities (H _ EO)	0.321 ***	0.081	3.958	0.000	1.354
L _ Entertainment Opportunities (L _ EO)	−0.146 **	0.055	−2.652	0.008	1.382
H _ Family Life (H _ FL)	0.394 ***	0.056	6.979	0.000	1.429
L _ Family Life (L _ FL)	−0.333 ***	0.092	−3.626	0.000	1.148
H _ Education Conditions (H _ EC)	0.118 *	0.059	2.012	0.045	1.264
L _ Education Conditions (L _ EC)	0.457	0.245	1.866	0.063	1.167

Notes: * *p* < 0.05, ** *p* < 0.01, *** *p* < 0.001.

## Data Availability

The dataset used in this research are available upon request from the corresponding author.
